# Aneuploid abortion correlates positively with MAD1 overexpression and miR-125b down-regulation

**DOI:** 10.1186/s13039-021-00538-1

**Published:** 2021-04-26

**Authors:** Juan Zhao, Hui Li, Guangxin Chen, Lijun Du, Peiyan Xu, Xiaoli Zhang, Min Xie, Tiansheng Cao, Haibo Li

**Affiliations:** 1 Department of the Clinical Laboratory, Affiliated Huadu Hospital, Southern Medical University (People’s Hospital of Huadu District), Guangdong, 510800 China; 2 Department of Internal Medicine, Affiliated Huadu Hospital, Southern Medical University (People’s Hospital of Huadu District), Guangdong, 510800 China; 3 Department of Obstetrics and Gynaecology, Affiliated Huadu Hospital, Southern Medical University (People’s Hospital of Huadu District), Guangdong, 510800 China; 4 Central Laboratory of Birth Defects Prevention and Control, Ningbo Women & Children’s Hospital, Ningbo, 315000 Zhejiang China; 5 Surgical Department, Affiliated Huadu Hospital, Southern Medical University (People’s Hospital of Huadu District), Guangdong, 510800 China

**Keywords:** Mir125b, MAD1, BUB3, Aneuploid abortion

## Abstract

**Background:**

Aneuploidy is the most frequent cause of early-embryo abortion. Any defect in chromosome segregation would fail to satisfy the spindle assembly checkpoint (SAC) during mitosis, halting metaphase and causing aneuploidy. The mitotic checkpoint complex (MCC), comprising MAD1, MAD2, Cdc20, BUBR1 and BUB3, plays a vital role in SAC activation. Studies have confirmed that overexpression of MAD2 and BUBR1 can facilitate correct chromosome segregation and embryo stability. Research also proves that miR-125b negatively regulates MAD1 expression by binding to its 3′UTR. However, miR-125b, Mad1 and Bub3 gene expression in aneuploid embryos of spontaneous abortion has not been reported to date.

**Methods:**

In this study, embryonic villi from miscarried pregnancies were collected and divided into two groups (aneuploidy and euploidy) based on High-throughput ligation-dependent probe amplification (HLPA) and Fluorescence in situ hybridization (FISH) analyses. RNA levels of miR-125b, MAD1 and BUB3 were detected by Quantitative real-time PCR (qRT-PCR); protein levels of MAD1 and BUB3 were analysed by Western blotting.

**Results:**

statistical analysis (*p* < 0.05) showed that miR-125b and BUB3 were significantly down-regulated in the aneuploidy group compared to the control group and that MAD1 was significantly up-regulated. Additionally, the MAD1 protein level was significantly higher in aneuploidy abortion villus, but BUB3 protein was only mildly increased. Correlation analysis revealed that expression of MAD1 correlated negatively with miR-125b.

**Conclusion:**

These results suggest that aneuploid abortion correlates positively with MAD1 overexpression, which might be caused by insufficient levels of miR-125b. Taken together, our findings first confirmed the negative regulatory mode between MAD1 and miR-125b, providing a basis for further mechanism researches in aneuploid abortion.

## Introduction

Aneuploidy is a common and natural occurrence in early human embryos that causes a disordered cell cycle and retards embryonic growth and even causes miscarriage [1]. A systematic review and meta-analysis associated with human embryo chromosomes in 2011 reported that 73% of all human embryos contain aneuploid cells [2, 3]. Chromosomal instability (CIN) plays a crucial role in aneuploidy and tumorigenesis [4]. Ivan Y Iourov et al. proposed a mechanism designated as "chromohelkosis" with the meaning that structural chromosomal imbalances are likely to cause local instability ("wreckage") at the breakpoints, which results either in partial/whole chromosome loss (e.g., aneuploidy) or elongation of duplicated regions [5]. In addition, Franck Pellestor reported that chromothripsis and chromoanasynthesis may essentially result from lagging chromosome encapsulated in micronuclei or telomere attrition and end-to-end telomere fusion [6]. In this context, the spindle assembly checkpoint (SAC) is crucial to ensure chromosome segregation during mitosis. Aneuploidy is known as a frequent outcome of a defective mitotic checkpoint [7]. Any defect in kinetochore–spindle attachment between sister chromatids would fail to satisfy the SAC, which would halt metaphase until the defect is corrected.

The process of chromosome segregation during mitosis is exceptionally complicated, consisting of a series of cascade reactions, and multiple proteins are involved. Once all chromatids are bi-oriented in metaphase, the E3 ubiquitin-ligase anaphase-promoting complex/cyclosome (APC/C) catalyses proteasomal degradation of securin, an inhibitory chaperone of separase. Activated separase then cleaves the cohesion complex required for the physical linkage of sister chromatids. Owing to a loss of cohesion, the separated chromatids move to opposite spindle poles, and the cell enters anaphase [9, 10]. Furthermore, APC/C activity depends on the adaptor protein Cdc20 (cell division cycle 20) [11]. When the SAC interacts with APC/C, the latter remains inactive because Cdc20 is sequestered by Mad2 and BubR1/Bub3 in the form of a mitotic checkpoint complex (MCC). Premature anaphase progression is thus prevented by the 'wait-anaphase' signal generated from diffusible MCC [12].

MCC, a complex of MAD1, MAD2, Cdc20, BUB1 and BUB3, plays a vital role in SAC activation. One of the first events in SAC activation is recruitment of the adaptor protein Mad1 to the kinetochore. Mad1 is crucial for both Mad2 transport from the cytosol to the nucleus and its kinetochore localization [8]. According to the template model, Mad2 exists in two states: open/free (O-Mad2) and closed (bound to either Mad1 or Cdc20 (C-Mad2)). Studies have confirmed that reduced expression of Mad2 and Bub1 proteins is associated with spontaneous miscarriage but that MAD2 overexpression can facilitate correct chromosome segregation and embryo stability [13].

Many mitotic cell cycle regulators often have modified functions in meiosis that are important for the meiotic chromosome segregation programme. A noteworthy example of a regulatory pathway with increased roles in meiosis is the spindle checkpoint.

Research shows that the spindle checkpoint proteins Mad2, Bub1 and Bub3 have a more important role in meiosis than in mitosis. Mad2 cells exhibit enhanced chromosome nondisjunction in meiosis I and premature anaphase I onset. Loss of BUB1 or BUB3 in meiosis causes massive chromosome missegregation in meiosis II, and aneuploidy still occurred despite fewer cells with massive chromosome missegregation in meiosis I [14]

Research from S Bhattacharjya et al. shows that miR-125b negatively regulates MAD1 expression by binding to its 3′UTR [15]. miR-125b, MAD1 and BUB3 gene expression in aneuploidy embryos of spontaneous abortion has not been reported to date. This study was designed to investigate expression of miR-125b, MAD1 and BUB3 in embryonic villus cells from aneuploidy abortion embryos and further explore the mechanism of spontaneous abortion.

## Materials and methods

### Cohort

Embryonic villi from miscarried pregnancies (5–11 weeks of pregnancy, women aged 21–40 years old, with no toxic and harmful substance contact experience) were collected and preserved at − 80 °C from May 2018 to May 2019 in the Central Laboratory of Birth Defects Prevention and Control, Ningbo Women & Children's Hospital, Ningbo, Zhejiang, China. Villus tissues were obtained after uterine cleanup. Based on the results of chromosomal aneuploidy (aneuploidy and euploidy) detection, 100 embryonic villus samples were divided into two groups: 50 cases with chromosomal abnormalities (abnormal group) and 50 cases without chromosomal abnormalities (normal group). The Ethics Committee approved this study.

## Methods

### Aneuploidy detection by HLPA

Abnormalities of 24 chromosomes were detected using a modified MLPA (multiplex ligation-dependent probe amplification) assay named HLPA (high-throughput ligation-dependent probe amplification), which was carried out with a CNVplex detection kit (Genesky Technologies (Suzhou) Inc.). The main principle of this method is to use the high specificity of ligase to perform a set of hybridization and ligation reactions of target regions to distinguish that regions' ploidy. In the ligation step, sequence tags within the different lengths and different fluorescein dyes (PET, VIC, NED, and FAM) were introduced at the ends of the probes and then ligated to the target regions to obtain ligated products of different lengths. Then, universal primers labelled with fluorescent markers were used to amplify the concatenated products by PCR. After amplification, the PCR products were separated and detected by fluorescence capillary electrophoresis. The copy number of target regions was calculated by analysing the electrophoretogram's peak height; the detailed workflow refers to the study Chen, S et al. reported [16]. In this study, 170 (eight probes for each chromosome for Chr1-12 and Chr16-17; five probes for each chromosome for Chr13-15, Chr21-22 and ChrY; seven probes for each chromosome for Chr18-Chr20 and ChrX) pairs of probes targeting 24 chromosomes were designed for aneuploidy detection. The experimental steps were as follows. First, two μl gDNA (30 ng/μL) mixed with 1 μl probe mix (10 µM), 1.25 μl 4 × DNA lysis buffer and 5.75 μl DNA diluent was denatured for 2 min at 98 °C and then immediately placed on ice. Next, 2 μl 10 × ligation buffer, 0.5 μl ligase and 7.5 μl double-distilled water were added to the products of the first step for ligation as follows: 5 cycles of 94 °C for 1 min and 60 °C for three h, 94 °C for 2 min, and 72 °C for 10 min. Reactions were stopped by adding 20 μl of 20 mM EDTA. After the ligation reaction, 1 μl of ligation products, 10 μl of 2 × PCR Master Mix, 1 μl of primer mix and 8 μl of double-distilled water were mixed to perform multiplex PCR amplification. The PCR programme was as follows: 95 °C for 2 min; 5 cycles of 94 °C for 20 s, 62 °C for 40 s decreasing 1 °C per cycle, and 72 °C for 1.5 min; 27 cycles of 94 °C for 20 s, 57 °C for 40 s, and 72 °C for 1.5 min; 68 °C for 1 h; and cooling to 4 °C to stop the reaction. The last step was capillary electrophoresis and data analysis. The PCR products were diluted fivefold, and 1 μl of diluted product was mixed with 0.1 μl of LIZ 500 size standard (Applied Biosystems, Foster City, CA, USA) and 8.9 μl of Hi-DiTM formamide (Applied Biosystems) were denatured at 95 °C for 5 min; the fluorescently labelled products were separated using an ABI3130XL genetic analyser (Applied Biosystems). The data were analysed with GeneMapper software v4.1 (Applied Biosystems).

### Karyotype verification by FISH

To confirm the aneuploidy detection results by HLPA, fluorescence in situ hybridization (FISH) was performed according to Escudero, Abdelhadi, Sandalinas, & Munné, 2003 [17]. The DNA probes used for this study were purchased from Beijing GP Medical Technologies, Inc., P.R. China. DLEU2:13q14, CSP18:18p11.1-q11.1, DSCR2:21q22, CSP X: Xp11.1-q11.1 and CSP Y: Yp11.2-q11.2 was used to detect chromosome Y. were used to detect chromosomes 13, 18, 21, X and Y, respectively. In the fixation step, embryonic villus cells were fixed in methanol/acetic acid (3:1), after which eight drops of fixed villus cells were placed on each slide. Once dried, the slides were washed twice in 2 × standard saline citrate (SSC, Vysis Inc.) at room temperature for 3 min each. The slides were dehydrated in an ethanol series (70%, 85% and 100%) for 2 min each and dried at room temperature in a slanted position. For denaturation, the dried slides were placed in 5 nmol/L dithiothreitol (DDT, Sigma) and 1% Triton X-100 solution (Sigma) at 37 °C for 13 min. After that, the slides were denatured in 70% formamide (Sigma) and 2 × SSC solution for 5 min at 71 °C. The slides were rewashed in 2 × SSC and dehydrated in an ethanol series. Before hybridization, the DNA probes mixed with hybridization buffer were immersed in a water bath for 5 min at 72 °C for denaturation. The denatured probe was then applied to each glass slide containing fixed villus cells and hybridized overnight at 37 °C. The slides were washed in 0.7 × SSC at 71 °C for 2 min, dried at room temperature, counterstained with DAPI in Antifade solution and covered with glass coverslips for analysis.

### Quantitative real-time PCR

Total RNA was isolated using TRIzol (Invitrogen) according to the manufacturer's protocol. Five micrograms of isolated RNA was treated with DNase (Promega, Madison, WI, USA), and 1 µg of DNase-treated RNA was used for cDNA preparation using random hexamers (Invitrogen) and MMLV-RT (Promega). To obtain miRNA, 200 ng of isolated RNA was used for cDNA preparation in a master mix containing stem-loop primers specific for the desired miRNAs (Sigma), dNTPs (Invitrogen) and MMLV-RT (Promega). Real-time PCR was performed using the 7500 Fast Real-Time PCR System (Applied Biosystems) with Power SYBR Green PCR Master Mix (Applied Biosystems). The comparative threshold cycle method (△△Ct) was used to quantify relative amounts of product transcripts using GAPDH (for mRNA) and U6 (for miRNA) as endogenous reference controls. The primer sets used for MAD1, BUB3 and GAPDH are listed in Supplementary Table 1. Fold activation values were calculated as the mean of the results of independent experiments (Table 1).

**Table 1:** Primers of miR-125b, MAD1 and BUB3

Primer name	Primer sequence
MAD1	F:GCCAGAAACAAAGAGCAGACAT; R:GACCTTCAACCTGAGCGTGT
BUB3	F:GAGTGGCGAGTAGTGGAAACG; R:AGGAGACAAGCAGGAACTGG
GAPDH	F:CCTCAACGACCACTTTGTCA; R:TCTTCCTCTTGTGCTCTTGCT
miR-125b	F: TCCCTGAGACCCTA; R: CAGTGCGTGTCGTGGAGT
U6	F:CTCGCTTCGGCAGCACA; R:AACGCTTCACGAATTTGCGT

### Western blotting and antibodies

Whole-cell lysates were obtained, and equal amounts of protein were resolved by SDS/PAGE (8–12% gel) and transferred to a PVDF membrane (Millipore, Billerica, MA, USA). The various primary antibodies used include mouse monoclonal anti-Mad1 (Millipore), mouse monoclonal anti-BUB3 (Cell Signalling Technology, Beverly, MA, USA), and mouse monoclonal anti-β-actin (Sigma). Bands were detected using Super Signal West Pico chemiluminescent substrate (Thermo Scientific, Rockford, IL, USA) after treatment with an HRP-conjugated secondary antibody (Sigma).

### Statistical analysis

SPSS 17.0 (SPSS Inc., Chicago, IL, USA; http://www.spss.com) was used to perform the Mann–Whitney t-test to determine significant differences between individual groups (normal and abnormal) for miR-125b, Mad1 and BUB3 expression. Statistically significant differences were defined by two-sided *p* < 0.05.

## Results

### Karyotype analysis

**Fig. 1:**
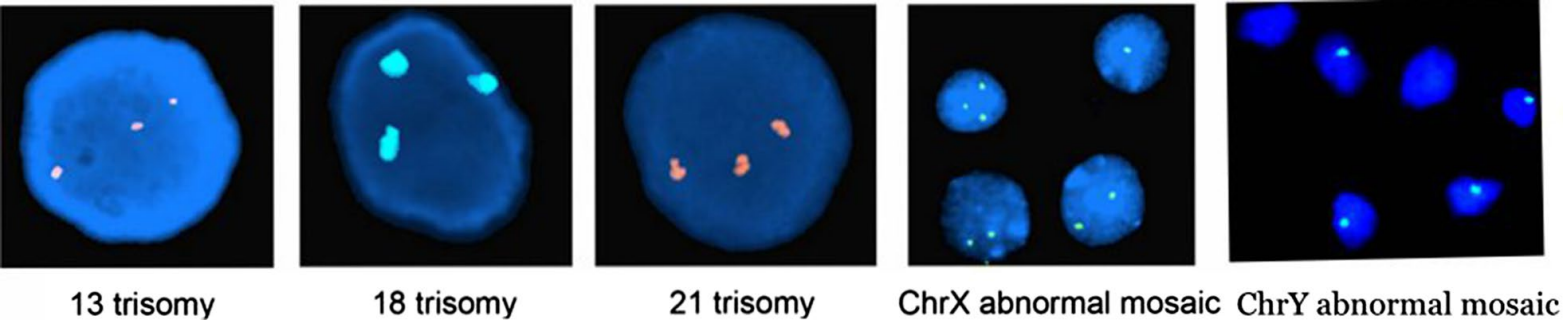
FISH verification of 13/18/21/X/Y chromosomal abnormalities. Red: 13 trisomy and 21 trisomy; Green: 18 trisomy, ChrX and ChrY abnormal mosaic; Blue: DAPI

Villi tissues were obtained after uterine cleanup. Chromosomal aneuploidy was detected by HLPA, fifty cases of chromosomal normalities were detected, including twenty-four cases with karyotype 46, XY and twenty-six of them are 46, XX. Fifty cases of chromosomal abnormalities were detected, as listed in Table 2. To confirm the aneuploidy detection results by HLPA, we further carried out FISH, and the results are shown in Fig. 1.

**Table 2:** 50 cases of chromosomal abnormalities by HLPA

Karyotype	Number of cases	Karyotype	Number of cases	Karyotype	Number of cases
46,XX,add(1)(p36)	1	47,XY,+14	1	47,XX,+12	2
47,XY,+2	2	47,XX,+14	1	47,XY,+13	2
48,XX,+2,+8	1	47,XX,+15	2	47,XX,+13	1
47,XY,+3	1	47,XY,+16	2	48,XXX,+13	1
47,XX,+4	1	47,XX,+16	8	47,XX,+22	3
46,XY,del(4)(p16)	1	47,XY,+18	3	45,X	6
47,XY,+6	2	48,XX,+11,+20	1	mos 47,XXX/46,XY/45, X	1
47,XX,+10	1	47,XY,+21	2	69,XXY	1
47,XX,+11	1	47,XY,+22	2

### Quantitative real-time PCR analysis of miR-125b, MAD1 and BUB3 RNA levels

**Fig. 2:**
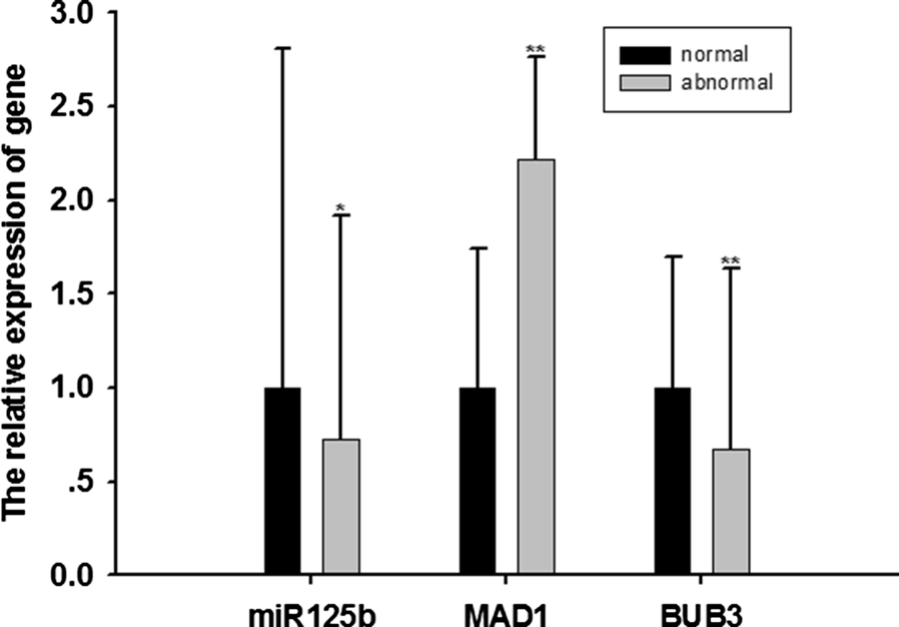
QRT-PCR analysis of miR-125b, MAD1 and BUB3 expression in chromosome normal and abnormal groups. *p < 0.05, **p < 0.01

Relative mRNA expression of miR-125b and BUB3 was significantly down-regulated in the abnormal group compared to the normal group (*p* < 0.05), whereas MAD1 was significantly increased at the RNA level (*p* < 0.05) (Fig. 2). Figure 2: miR-125b was significantly down-regulated in the abnormal group compared to the normal group (*p* < 0.05). However, MAD1 gene expression in the abnormal group was significantly up-regulated compared to that in the normal group, and relative expression of the BUB3 gene in the abnormal group was lower than that in the normal group (*p* < 0.05).

### Western blotting analysis of MAD1 and BUB3 proteins

**Fig. 3:**
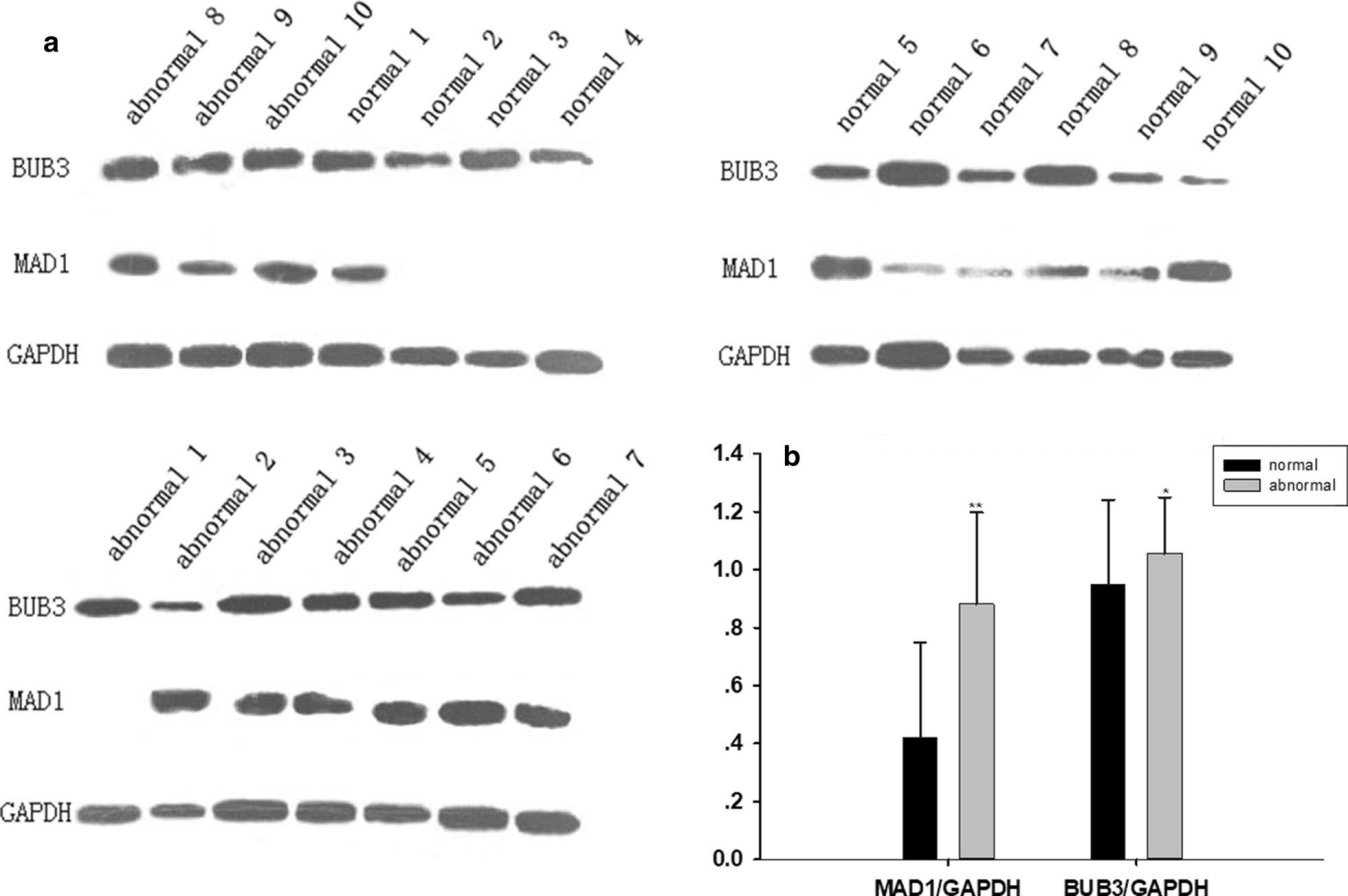
Western blotting analysis of relative expression of MAD1 and BUB3 proteins in chromosome normal and abnormal groups. GAPDH protein was used as an internal control. **a** WB diagram,** b** the protein gray value statistics.*p < 0.05, **p < 0.01

Relative expression of the BUB3 protein in the abnormal group was slightly up-regulated, while that of the MAD1 protein was significantly up-regulated (Fig. 3). BUB3 protein levels in the abnormal group were slightly up-regulated, while relative MAD1 protein expression was significantly up-regulated (*p* < 0.05).

## Discussion

Aneuploidy is the most frequent aetiology of miscarriage, and elucidating aneuploid molecular mechanisms is important for genetic consultation with women who experience abortion. The SAC is vital to ensuring the fidelity of chromosome segregation during mitosis, and aneuploidy can result in SAC failure. It is well known that MCC exerts an important influence on the whole SAC programme. MAD1, MAD2, Cdc20, BUBR1 and BUB3 have been confirmed to be important components of MCC. Among them, MAD2 and BUBR1 overexpression can facilitate correct chromosome segregation and embryo stability, though the expression levels of MAD1 and BUB3 in aneuploid abortion remain unknown.

According to the literature, miR-125b is a highly conserved miRNA that functions as a tumour suppressor or an oncogene depending upon the cellular context. Dandan Li and Jian Li’s manuscript pointed out that miR-125b was upregulated in unexplained recurrent spontaneous abortion (URSA) compared to the control group [18], In addition, the study by Fulu Dong et al. also proved that miR-125b-2 expression was significantly higher in RSA [19], which results were both opposite with our data, indicating that the expression of miR-125b may have heterogeneity in different patients, and might be differentially expressed between different subtypes or influenced by other unclear factors. Interestingly, the level of miR-125b was reported to vary at different gestational age, which was significantly increased in the preimplantation period, and was downregulated in the implantation period and the post-implantation period [20]. S Bhattacharjya et al. reported that miR-125b negatively regulates MAD1 expression by binding to its 3′UTR; miR-125b up- or down-regulation may give rise to CIN, leading to accelerated proliferation and cell death12 [21]. Indeed, previous studies have reported that Mad1 expression is increased in cancer cells and that its high expression correlates with cellular proliferation [22]. At the molecular level, Mad1-bound and free Mad2 are both essential to maintain the SAC. Moreover, excess MAD1 was superior to CDC20 in binding with free MAD2, whereas CDC20 can bind to and activate APC/C. All these factors lead to rapid mitotic exit and SAC abolishment [23, 24].

Two crucial regulators of the cell cycle, cohesin and the SAC, share standard regulators to coordinate faithful chromosome segregation and orchestrate meiotic progression, and research shows that the cohesin release factor Wapl interacts with Bub3 to mediate SAC function in oocyte meiosis I. Wapl regulates Bub3 in an unknown manner, which requires further investigation [19]. Other research shows that Bub3 is crucial for correcting attachment errors in meiosis. In addition, depletion of Bub3 results in reduced levels of kinetochore-localized Ipl1 and concomitant massive chromosome missegregation caused by incorrect chromosome-spindle attachment. Depletion of Bub3 also results in shorter metaphase I and metaphase II due to premature localization of protein phosphatase 1 (PP1) to kinetochores, which antagonizes Ipl1-mediated phosphorylation. A new role for the Bub1–Bub3 pathway in maintaining the balance between kinetochore localization of Ipl1 and PP1, a balance essential for accurate meiotic chromosome segregation and timely anaphase onset, has been proposed [25].

In this study, relative expression of the BUB3 gene was lower in the abnormal group than in the normal group. However, relative expression of BUB3 protein was slightly up-regulated. in the abnormal group. Overall, the relationship between BUB3 gene expression, the regulatory pathway and aneuploidy requires further investigation.

In this study, miR-125b levels decreased significantly in the abnormal group compared to the normal group, though the mRNA and protein levels of MAD1 were higher. Moreover, MAD1 expression correlated negatively with miR-125b. Taken together, we hypothesize that insufficient miR-125b increases levels of MAD1 expression, which results in decreased free Mad2 and activated APC/C. Such dysregulation does not satisfy the SAC and leads to accumulation of aneuploid cells in the embryo, which ultimately cause miscarriage through the child-to-mother communication mechanism.

The elaborate regulatory mechanism between miR-125b and MAD1 in aneuploid-induced spontaneous abortion warrants further exploration.

## Supplementary Information

**Additional file 1: Table S1.** Relative expression of miR-125b, MAD1 and BUB3 was calculated by using original qRT-PCR data. **Figure. S1.** The related indexes of Table S1 were plotted using Sigmaplot software. **Table S2.** The data were analysed according to the grey value of the WB signal of MAD1 and BUB3 protein expression. **Figure. S2.** WB of MAD1 and BUB3. The related indexes of Table S2 were plotted using Sigmaplot software.

## Data Availability

The CDS can be mainly obtained from NCBI: https://pmlegacy.ncbi.nlm.nih.gov/gene/?term=MAD1; https://pmlegacy.ncbi.nlm.nih.gov/gene/?term=BUB3; The DNA sequence can be mainly obtained from UCSC:http://genome.ucsc.edu/cgi-bin/hgTracks; Primer design uses Primer5.

## Abbreviations

PCR: Polymerase chain reaction
qRT-PCR: Quantitative real-time PCR
HLPA: High-throughput ligation-dependent probe amplification
FISH: Fluorescence in situ hybridization
WB: Western blotting
CNV: Copy number variation
SAC: Spindle assembly checkpoint
MCC: Mitotic checkpoint complex
CIN: Chromosomal instability
MLPA: Multiplex ligation-dependent probe amplification

## References

[1] Vanneste E, Melotte C, Voet T (2011). PGD for a complex chromosomal rearrangement by array comparative genomic hybridization. Hum Reprod.

[2] van Echten-Arends J, Mastenbroek S, Sikkema-Raddatz B (2011). Chromosomalmosaicism in human preimplantation embryos: a systematic review. Hum Reprod Update.

[3] Mantikou E, Wong KM, Repping S (1822). Mastenbroek S (2012) Molecular origin of mitotic aneuploidies in preimplantation embryos. Biochim Biophys Acta Mol Basis Dis.

[4] Vishwakarma R, McManus KJ (2020). Chromosome instability; implications in cancer development, progression, and clinical outcomes. Cancers (Basel).

[5] Iourov IY, Vorsanova SG, Yurov YB (2020). The cytogenomic "theory of everything": chromohelkosis may underlie chromosomal instability and mosaicism in disease and aging. Int J Mol Sci.

[6] Pellestor F (2019). Chromoanagenesis: cataclysms behind complex chromosomal rearrangements. Pellest Mol Cytogenet.

[7] Kops GJ, Weaver BA, Cleveland DW (2005). On the road to cancer: aneuploidy and the mitotic checkpoint. Nat Rev Cancer.

[8] Sironi L, Melixetian M, Faretta M (2001). Mad2 binding to Mad1and Cdc20, rather than oligomerization, is required for the spindle checkpoint. EMBO.

[9] Nasmyth K (2002). Segregating sister genomes: the molecular biology of chromosome separation. Science.

[10] De Antoni A, Pearson CG, Cimini D (2005). The Mad1/Mad2 complex as a template for Mad2 activation in the spindle assembly checkpoint. Curr Biol.

[11] Yu H (2007). Cdc20: a WD40 activator for a cell cycle degradation machine. Mol Cell.

[12] Musacchio A, Salmon ED (2007). The spindle-assembly checkpoint in space and time. Nat Rev Mol Cell Biol.

[13] Shi Q, Hu M, Luo M (2011). Reduced expression of Mad2 and Bub1 proteins is associated with spontaneous miscarriages. Mol Hum Reprod.

[14] Cairo G, MacKenzie AM, Lacefield S (2020). Differential requirement for Bub1 and Bub3 in the regulation of meiotic versus mitotic chromosome segregation. J. Cell Biol..

[15] Bhattacharjya S, Nath S, Ghose J, Maiti GP (2013). miR-125b promotes cell death by targeting spindle assembly checkpoint gene MAD1 and modulating mitotic progression. Cell Death Differ.

[16] Chen S, Liu D, Zhang J (2017). A copy number variation genotyping method for aneuploidy detection in spontaneous abortion specimens. Prenat Diagn.

[17] Escudero T, Abdelhadi I, Sandalinas M (2003). Predictive value of sperm fluorescence in situ hybridization analysis on the outcome of preimplantation genetic diagnosis translocations. Fertil Steril.

[18] Li D, Li J (2016). Association of miR-34a-3p/5p, miR-141-3p/5p, and miR-24 in decidual natural killer cells with unexplained recurrent spontaneous abortion. Med Sci Monit.

[19] Dong F (2014). Genome-wide miRNA profiling of villus and decidua of recurrent spontaneous abortion patients. Reproduction.

[20] Chen C, Zhao Y, Yu Y (2016). MiR-125b regulates endometrial receptivity by targeting MMP26 in women undergoing IVF-ET with elevated progesterone on HCG priming day. Sci Rep.

[21] Bhattacharjya S, Nath S, Ghose J, Maiti GP (2013). miR-125b promotes cell death by targeting spindle assembly checkpoint geneMAD1and modulating mitotic progression. Cell Death Differ.

[22] Iwanaga Y, Jeang KT (2002). Expression of mitotic spindle checkpoint protein hsMAD1 correlates with cellular proliferation and is activated by a gain-of-function p53 mutant. Cancer Res.

[23] Chung E, Chen RH (2002). Spindle checkpoint requires Mad1-bound and Mad1-free Mad2. Mol Biol Cell.

[24] Ryan SD, Britigan EM, Zasadil LM (2012). Up-regulation of the mitotic checkpoint component Mad1 causes chromosomal instability and resistance to microtubule poisons. Proc Natl Acad Sci USA.

[25] Zhou C, Miao Y, Cui Z (2020). The cohesin release factor Wapl interacts with Bub3 to govern SAC activity in female meiosis I. Sci Adv.

