# Supplementary material for: Aneuploid abortion correlates positively with MAD1 overexpression and miR-125b down-regulation
Source: Mol Cytogenet. 2021 Apr 26;14:22. doi: 10.1186/s13039-021-00538-1 (PMC8074413; doi:10.1186/s13039-021-00538-1)
Supplement: Supplementary file 1 — Additional file 1: Table S1. Relative expression of miR-125b, MAD1 and BUB3 was calculated by using original qRT-PCR data. Figure. S1. The related indexes of Table S1 were plotted using Sigmaplot software. Table S2. The data were analysed according to the grey value of the WB signal of MAD1 and BUB3 protein expression. Figure. S2. WB of MAD1 and BUB3. The related indexes of Table S2 were plotted using Sigmaplot software. [file 13039_2021_538_MOESM1_ESM.docx]

| **Sample** | **Genes** | **ΔCt** | **ΔΔCt** | **2（-ΔΔCt）** | **SE** | **p** |
| --- | --- | --- | --- | --- | --- | --- |
| Abnormal Group | miR125b | 4.57465 | 0.454533 | 0.72974635 | 1.18449775 | 0.023347657 |
| Normal Group | miR125b | 4.120117 |  | 1 | 1.80586265 |  |
| Abnormal Group | MAD1 | 8.138 | -0.59423 | 1.501 | 1.215 | 7.9328E-15 |
| Normal Group | MAD1 | 8.732 |  | 1 | 1.259 |  |
| Abnormal Group | BUB3 | 5.393 | 0.518272 | 0.698 | 0.813 | 0.00513 |
| Normal Group | BUB3 | 4.875 |  | 1 | 0.799 |  |

Table S1：miR125b was significantly down-regulated in the abnormal group compared to normal group(P<0.05)；MAD1 gene in abnormal group was significantly up-regulated than that in normal group, and the relative expression of BUB3 gene in abnormal group was lower than that in normal group (p <0.05).

The relative expression of miR125b,MAD1and BUB3 indexes calculated by using QRT-PCR original data .


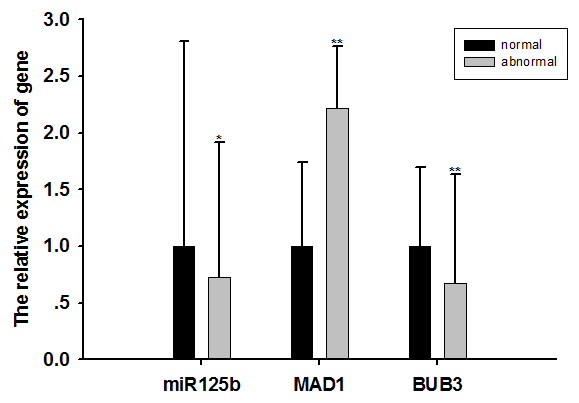


Figure S1: The relative expression of miR125b, MAD1 and BUB3

Use related indexes of Table S1 , plotted by Sigmaplot software

| **Sample** | Normal Group | | | Abnormal Group | | |  |
| --- | --- | --- | --- | --- | --- | --- | --- |
| **Protein** | mean | SD | SE | mean | SD | SE | p |
| BUB3/GAPDH | 0.947228 | 0.508136 | 0.293372 | 1.05567 | 0.336027 | 0.194005 | 0.0241317 |
| MAD1/GAPDH | 0.419518 | 0.570243 | 0.32923 | 0.881839 | 0.547293 | 0.315979 | 0.000175 |

Table S2：The data analyzed according to the gray value of the WB strip of MAD1 and BUB3 protein expression.

**
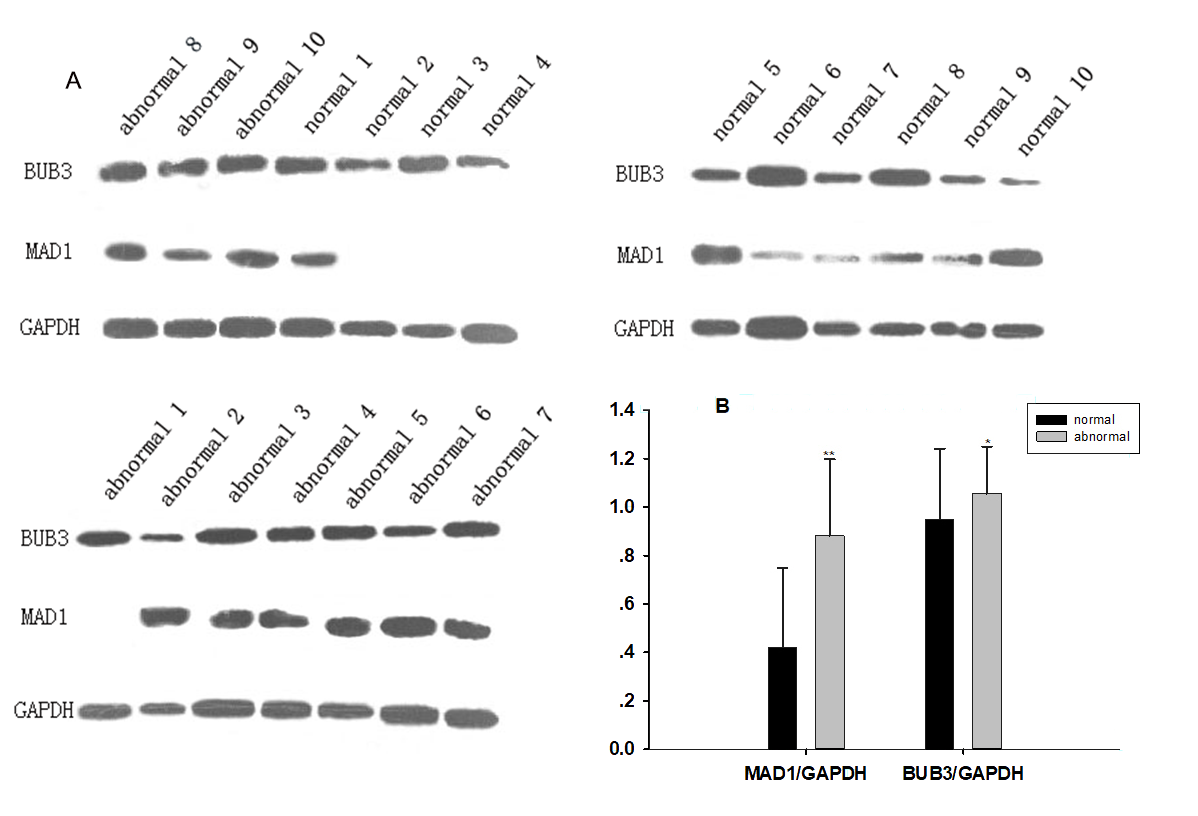
**

Figure S2:BUB3 protein in abnormal group was slightly up-regulated,while the relative expression of MAD1 protein in abnormal group was significantlys up-regulated.while the relative expression of MAD1 protein in abnormal group was significantly up-regulated (P<0.05).

WB glue map of MAD1 and BUB3;Use related indexes of Table S2 , plotted by Sigmaplot software .
